# Transition Metal Substituted Barium Hexaferrite-Modified Electrode: Application as Electrochemical Sensor of Acetaminophen

**DOI:** 10.3390/molecules27051550

**Published:** 2022-02-25

**Authors:** Claudia Patricia Granja-Banguera, Daniel Gerardo Silgado-Cortázar, Jimmy Alexander Morales-Morales

**Affiliations:** Chemistry and Biotechnology Research Group (QUIBIO), Faculty of Basic Sciences, Campus Pampalinda, Universidad Santiago de Cali, Cali 760035, Colombia; claudia.granja00@usc.edu.co (C.P.G.-B.); daniel.silgado00@usc.edu.co (D.G.S.-C.)

**Keywords:** nanopowder, catalyst, sensor, electro-oxidation

## Abstract

This study used substituted barium hexaferrites, which were previously prepared and reported by the authors, to detect acetaminophen by the modification of a conventional glassy carbon electrode (GCE), which led to promising results. The synthesis of this electrode-modifying material was conducted using a citrate sol gel process. A test synthesis using glycerin and propylene glycol revealed that glycerin produced a better result, while less positive anodic potential values were associated with the electrooxidation of N-acetyl-p-aminophenol (NAP). Excellent electroactivity was exhibited by the cobalt-substituted barium-hexaferrite-nanomaterial-modified electrode. A good linear relationship between the concentration and the current response of acetaminophen (paracetamol) was obtained with a detection limit of (0.255 ± 0.005) µM for the Ba_1.0_Co_1.22_Fe_11.41_O_18.11_ GCE, (0.577 ± 0.007) µM for the Ba_1.14_Cu_0.82_Fe_11.65_O_18.02_ GCE, and (0.595 ± 0.008) µM for the bare GCE. The levels of NAP in a real sample of urine were quantitatively analyzed using the proposed method, with recovery ranges from 96.6% to 101.0% and 93.9% to 98.4% for the modified electrode with Cobalt-substituted barium hexaferrites (CoF_M_) and Copper-substituted barium hexaferrites (CuF_M_), respectively. These results confirm the high electrochemical activity of Ba_1.0_Co_1.22_Fe_11.41_O_18.11_ nanoparticles and thus their potential for use in the development of sensing devices for substances of pharmaceutical interest, such as acetaminophen (NAP).

## 1. Introduction

N-acetyl-p-aminophenol (NAP) (i.e., acetaminophen) is used in the manufacture of tinctures and photography and as an analgesic and febrifuge; consequently, it is incorporated into the environment via multiple routes. Occupational exposure can occur via inhalation or dermal absorption at sites of manufacturing or use, although its main source of exposure results from its widespread use as an analgesic. The most-reported route of contamination is via unchanged excretion in urine and feces in medical and domestic settings; consequently, this commonly used medication is a potential water pollutant. Therefore, developing an analytical approach for the simple and precise quantification of this analyte is particularly challenging.

Various techniques have been proposed for the detection of NAP [1,2,3,4,5,6,7,8,9,10,11]. Electroanalytical methods have been used in the field of drug analysis, and in recent decades a variety of nanomaterials have been used to modify the surface of the electrodes to efficiently determine electroactive species of interest and improve the sensitivity of electrochemical sensors [12,13]. In modern voltammetry, chemically modified electrodes are now a developed area in which they are widely recognized for their superior properties of selectivity, sensitivity, and in situ performance.

The modification of an electrode’s surface is intended to increase the electroactive area and active adsorption sites and enhance the selective interactions between the modified electrode and the analyte. Electrode modifiers have been widely used to improve the electroactive surface area, increase the stability, and reduce the charge-transfer resistance of electrodes in comparison with bare electrodes. Electrodes can be modified using magnetic nanoparticles; by increasing the sensitivity and stability of the sensors, they can help in detecting trace levels of different analytes [14].

The unique physical and chemical properties of magnetic nanoparticles have made them an attractive research focus. In the field of electrochemistry, particularly electroanalysis, they are utilized extensively as functional materials due to their high surface area, mass transport, catalytic effect, and control over the local environment. Barium-based ferrites are attractive materials of interest that have emerged due to their interesting physical, chemical, magnetic, and electrical properties. Nano-ferrite-based sensors have many advantages, including high sensitivity and signal-to-noise ratios, low determination limits, and short analysis time [15].

One important group of magnetic oxides is M-type hexagonal ferrites, e.g., barium ferrite; these are used in different sensor technologies on account of their specific favorable properties, including low cost, corrosion-resistant chemical stability and large magnetocrystalline anisotropy [16,17,18]. M-type barium ferrite has a molecular formula BaFe_12_O_19_ and a crystalline structure of magneto plumbite [19]. Adequate groundwork has been laid regarding the effect of different elements on the characteristics of BaFe_12_O_19_ [20]. However, investigations into substituted BaFe_12_O_19_ and the effect of other elements on the structure of M-type hexagonal ferrite and its electrochemical properties remains the subject of increasing research. Various methods of synthesizing M-type barium ferrite powder particles have been developed [21,22,23,24,25,26,27,28,29,30]. For example, the widely used combustion method is reliable, fast, and efficient, and when the process includes transition-metal chlorides, ferrites with satisfactory particle size can be obtained [31,32,33,34,35,36,37,38].

BaFe_12_O_19_ nanoparticles have been utilized in electroanalysis due to their considerable electrocatalytic activity [39,40]. On this basis, transition-metal-substituted barium M-type hexagonal ferrite particles with Fe/Ba and a molar ratio of 4 could be used for the electrooxidation processes. Ferrite substituents are especially crucial since they determine variations in physical, magnetic, and electrical transport properties, [38]. The authors recently prepared substituted spinel ferrites of cobalt and copper with potential application in the design/elaboration of metabolite-sensing surfaces for medical applications [41]. Employing the same synthesis method, cobalt- and copper-substituted M-type hexagonal ferrite particles with Fe/Ba and a molar ratio of 4 were synthesized using the sol–gel combustion technique.

To the best of the authors’ knowledge, there have been no reports on the application of cobalt- and copper-substituted M-type barium ferrite powder particles with Fe/Ba (molar ratio of 4) in the electrooxidation processes of acetaminophen. Therefore, the present investigation examines the electrochemical properties of M-type barium ferrite powder particles with an Fe/Ba ratio of 4 in the oxidation of NAP.

## 2. Results and Discussion

### 2.1. Characterization of Transition-Metal-Substituted Barium Hexaferrites

#### 2.1.1. X-ray Diffraction Analysis

The crystallographic structure of the samples was determined using X-ray diffraction (XRD). Table 1 presents the diffraction patterns of the synthesized cobalt- and copper-substituted M-type barium ferrite powder particles and their corresponding planes at 2(θ) values of 30.24° (110), 30.20° (107), 37.12° (203), 40.30° (205), 42.35° (206), 55.10° (217), 56.58° (2011), and 63.16° (220) (peaks marked “F”); these include hexaferrites with preferred orientation along the 30.20° (107) and 34.10° (114) planes [20], which are similar to those in a previously published study [39].

**Figure 1:**
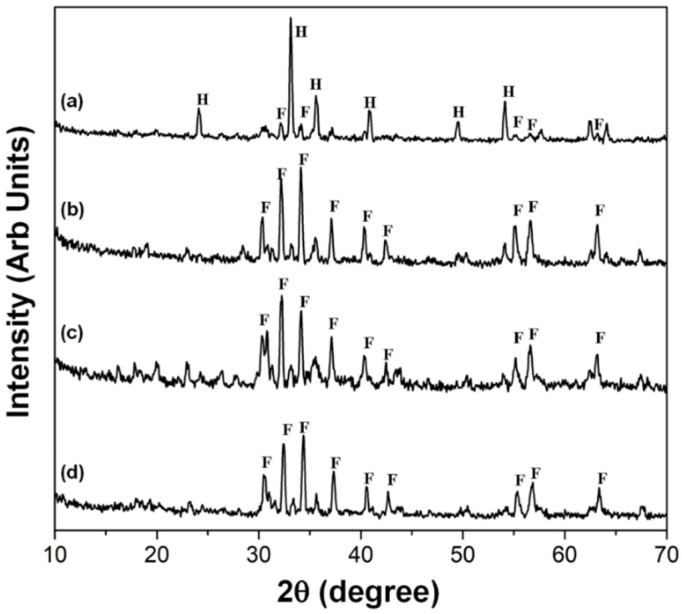
XRD patterns of M-type barium hexaferrite: (**a**) unsubstituted, (**b**) cobalt substituted, (**c**) copper substituted, and (**d**) unsubstituted. Nanomaterials were sintered at 950 °C for 3 h using (**a**) propylene glycol and (**b**–**d**) glycerin, respectively. F = M-type barium ferrite and H = hematite.

The synthesis methodology followed that of a previously developed procedure [20]. The effect of two alcohols (propylene glycol and glycerin) was evaluated, and the results in the crystal structure are shown in Figure 1a. Propylene glycol led to the formation of the hematite phase in a higher proportion when compared with unsubstituted barium ferrite. Figure 1b–d illustrates the effect of adding glycerol, which promoted the formation of the M-type barium ferrite phase in a greater proportion when compared to the hematite phase. In Figure 1d, there is evidence of the formation of unsubstituted barium ferrites, where signals (the peaks marked “F”) correspond to the diffraction pattern of the ferrite. While the signals (the peaks marked “H”) correspond to hematite phases, the formation of impurities, e.g., hematite (α−Fe_2_O_3_), is lower compared with (b) cobalt- and (c) copper-substituted M-type barium ferrite.

The peaks in Figure 1b,c correlate well with the data reported for M-type ferrites substituted with cobalt [35] and copper [22]. The XRD patterns of the ferrite samples reveal characteristic diffraction peaks corresponding to the structure of the M-type barium ferrite; the point group of P63/mmc indicates that there is no transformation of the crystal structure, which, after substitution with copper or cobalt ions, remains as hexagonal magneto plumbite. Figure 1b–d exhibits soft peaks that correspond to the hematite phase. During sintering at 950 °C, there was an increase in the fraction of M-type barium ferrite, while the amount of hematite remained unchanged [38,39,40]. This is the reason for the endurance of the hematite phase (see Figure 1b–d).

These results reveal that glycerol facilitated the formation of hexagonal M-type ferrites. However, its function as an agglomeration obstructer was not confirmed under the experimental conditions of this study.

#### 2.1.2. Raman Spectroscopy

**Figure 2:**
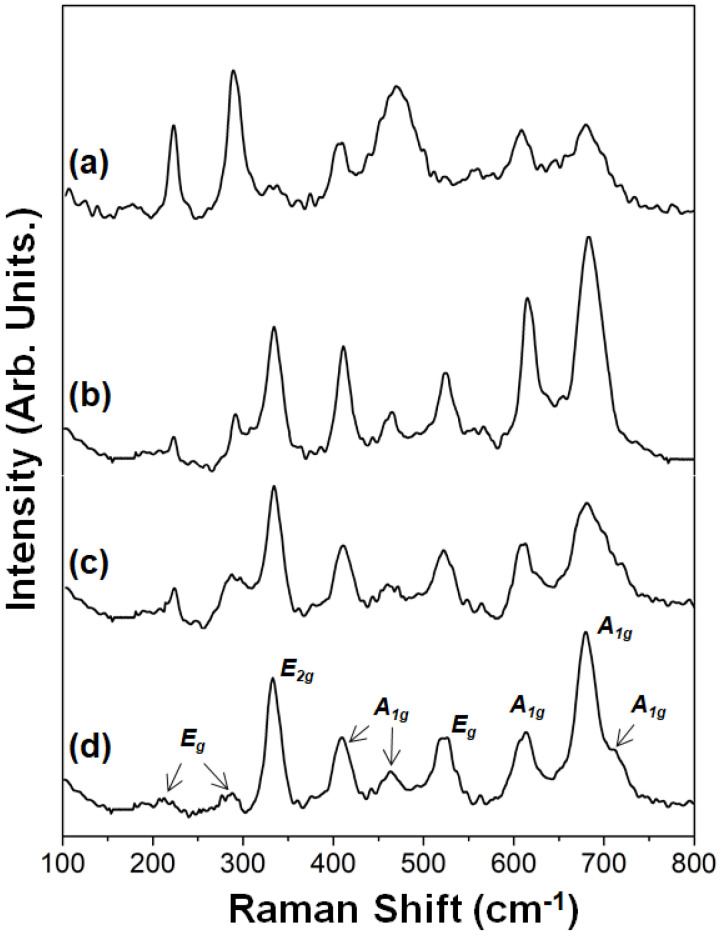
Raman spectra and E_g_, E_2g_, A_1g_ active modes of M-type barium hexaferrite: (**a**) unsubstituted, (**b**) cobalt substituted, (**c**) copper substituted, and (**d**) unsubstituted. The nanomaterials were sintered at 950 °C for 3 h using (**a**) propylene glycol and (**b**–**d**) glycerin, respectively.

Figure 2 presents the characteristic peaks of different M-type barium ferrites synthesized by the citrate sol–gel technique. These peaks were assigned based on the Raman spectral analysis of hexaferrites as reported by Kreisel et al. and Zhao et al. [39,40]. Figure 2a–d shows the differences/modifications resulting from the use of propylene glycol and glycerin, respectively. The appearance of a set of nine peaks (Figure 2d) was confirmed during the analysis of the Raman spectrum of the unsubstituted barium ferrite sample. Peaks at 610, 681, and 709 cm^−1^ were assigned to active A_1g_ modes that corresponded sequentially to octahedral (4f_2_), bipyramidal (2b), and tetrahedral (4f_1_) sites. Other peaks of active-mode A_1g_ were observed at frequencies of 411 and 456 cm^−1^, which corresponded to octahedral sites. An intense peak at 338 cm^−1^ was assigned to the E_2g_ active mode of the octahedral sites (12k), while the peaks at 215, 290, and 530 cm^−1^ corresponded to active-mode E_g_ [42]. Figure 2a reveals that the latter three peaks were more intense in the Raman spectrum for the sample of barium ferrite in propylene glycol.

The presence of the hematite phase in the sample could affect these peaks due to similar vibrational frequency between the phases. The most representative bands of hematite occurred at around 228 and 295 cm^−1^ [43,44]. The shifting of the Raman bands toward a lower wavenumber could be attributed to the influence of quantum confinement [45]. The above proposition indicates the presence of hematite phase in the M-type barium ferrite sample synthesized using propylene glycol. The displacements toward lower vibrational frequencies resulted due to copper and cobalt atoms inserted in the structure of barium ferrite. A similar signal pattern is presented in Figure 2b–d. However, in Figure 2a, the differences in the patterns of the observed peaks resulted from the effect of propylene glycol and the other phases that were present in the sample. According to the results of Raman analysis, it was confirmed that the synthesis of M-type barium ferrite substituted with copper and cobalt, separately, was adequate when glycerin was used.

#### 2.1.3. Field-Emission Scanning Electron Microscopy Analysis

**Figure 3:**
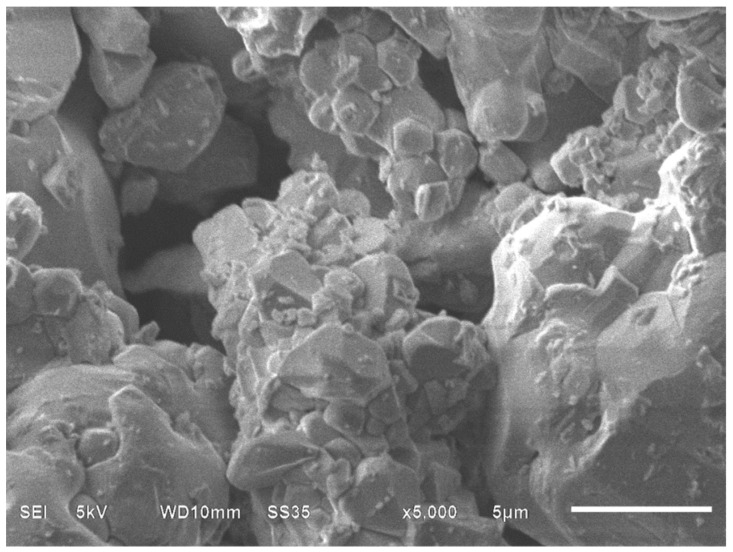
FESEM image of unsubstituted barium hexaferrite Ba_1.0_Fe_11.72_O_18.72_.

The morphology of the synthesized BaFe_12_O_19_ nanoparticles was determined using field-emission scanning electron microscopy (FESEM). An image of typical unsubstituted barium hexaferrite is shown in Figure 3. This nanomaterial presented sizes in the range of 200–500 nm, and its high agglomeration made it difficult to accurately determine the particle size and morphology of the image. Additionally, there was a moderate presence of irregular-shaped particles.

The chemical composition of this sample was determined using an Atomic absorption spectroscopy (AAS) device as Ba_1.0_Fe_11.72_O_18.72_, Ba_1.0_Co_1.22_Fe_11.41_O_18.11_, and Ba_1.14_Cu_0.82_Fe_11.65_O_18.02_, which were very close to the nominal composition.

### 2.2. Characterization by Electrocatalysis

#### 2.2.1. Electrochemical Characterization of Electrodes

**Figure 4:**
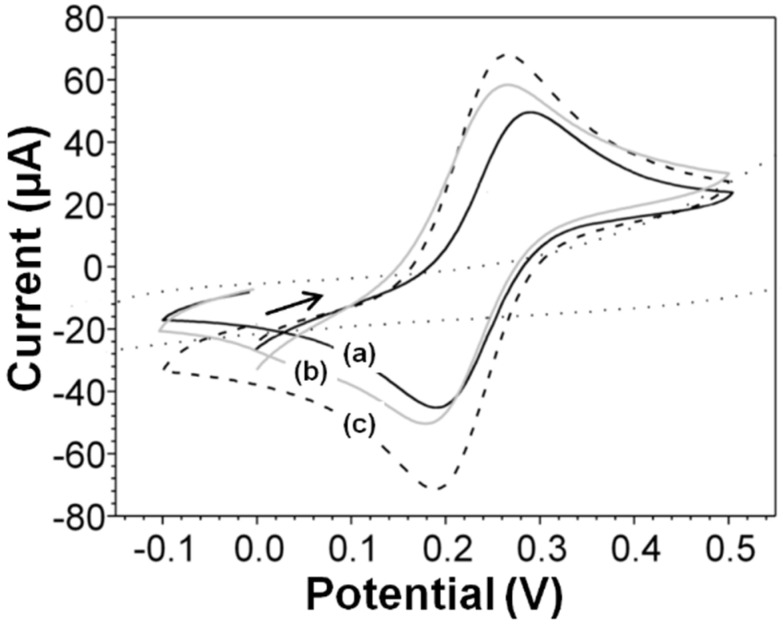
Cyclic voltammograms of (a) bare GCE dashed line, (b) CuF_M_ GCE solid line and (c) CoF_M_ GCE dot line in 1 × 10^−4^ M K_3_[Fe(CN)_6_] in 0.1 M KCl solution at a scan rate of 0.1 Vs^−1^.

Figure 4 shows the electrochemical behavior of a nearly reversible electrode reaction of potassium ferricyanide (K_3_[Fe(CN)_6_]) on a modified and unmodified glassy carbon electrode (GCE) with copper- and cobalt-substituted barium hexaferrites.

The modified electrodes produced an increase in the peak redox current relative to the bare electrode. In Figure 4a,b, the GCE, the cyclic voltammogram demonstrated the oxidation and reduction peak potentials of 289 and 205 mV, respectively, at a scan rate of 0.1 Vs^−1^. Figure 4c presents the oxidation and reduction peak potentials at 264 and 197 mV, respectively, at a scan rate of 0.1 Vs^−1^. The changes in peak potential (17 mV) and peak current confirmed the effect of a larger electrode area of the CoF_M_ GCE.

A test performed under different scan rates revealed that the peak current varied linearly with respect to the square of the scan rate. Using the Randles–Ševčík equation [46], the areas of the GCE, CuF_M_ GCE, and CoF_M_ GCE were confirmed to be 0.073, 0.079, and 0.84 cm^2^, respectively.

#### 2.2.2. Electrochemical Response of NAP

**Figure 5:**
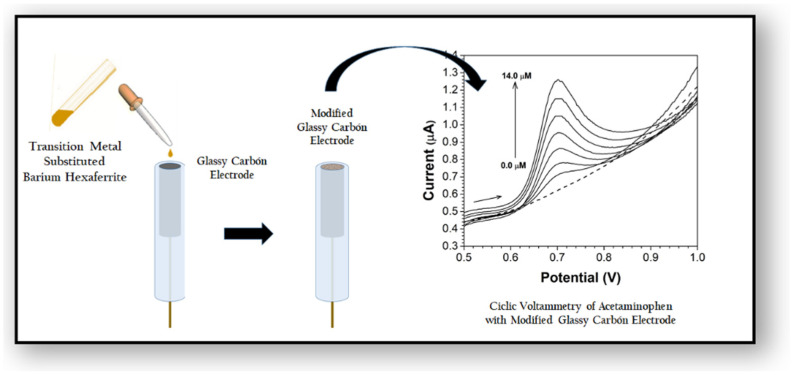
Schematic diagram of the electrode modification procedure and the electrochemical response of NAP at the electrode.

Figure 5 presents a schematic diagram summarizing the chemical modification of the electrode and the voltammetric response of the NAP analyte at the copper- and cobalt-substituted barium hexaferrite (bare and modified) GCEs (2 × 10^−3^ mol L^−1^ NAP in 0.1-M phosphate-buffered saline [PBS], pH = 2.50).

**Figure 6:**
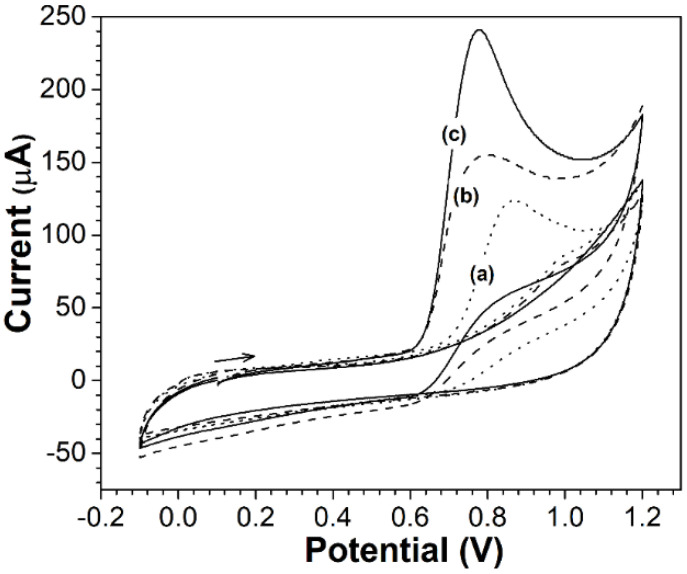
Cyclic voltammograms of 2 × 10^−3^ mol L^−1^ NAP in 0.1 M PBS (pH = 2.5) on (a) bare electrode dotted line, (b) CuF_M_ GCE dashed line, and (c) CoF_M_ GCE in solution, respectively, at a scan rate of 0.1 Vs^−1^.

The electrochemical properties of copper- and cobalt-substituted barium hexaferrites (bare and modified electrodes) were investigated using a cyclic voltammetry (CV) of 2.0-mM acetaminophen at 0.1 Vs^−1^, a potential window of −0.2–1.4 V in 0.1-M PBS and a pH of 2.50. Figure 6 shows the comparative cyclic voltammograms of the electrodes.

The electrochemical oxidation of acetaminophen produced higher current responses at the modified electrodes (CuF_M_ GCE and CoF_M_ GCE) than at the bare electrodes. This could be attributed to the presence of barium hexaferrite nanoparticles substituted with copper, which led to an enhancement in the electrical conductivity and porosity; this exposed the analyte to more active surface sites and an increase in mass transport [47]. Figure 6 shows a change in the value of the potential peak. The anodic peak potential at the modified electrodes was observed to be less positive than at the bare electrode, and an increase in the anodic peak current at the modified electrodes was also observed. Together, these facts indicate that the synthesized nanoparticles effectively catalyzed the electrochemical oxidation of NAP [48,49]. Concerning the barium ferrites in this study, the greatest effect occurred when using the hexaferrites substituted with cobalt rather than copper.

Table 1 contains details of the parameters determined in the cyclic voltammetric detection of acetaminophen on bare and modified electrodes.

**Figure 7:**
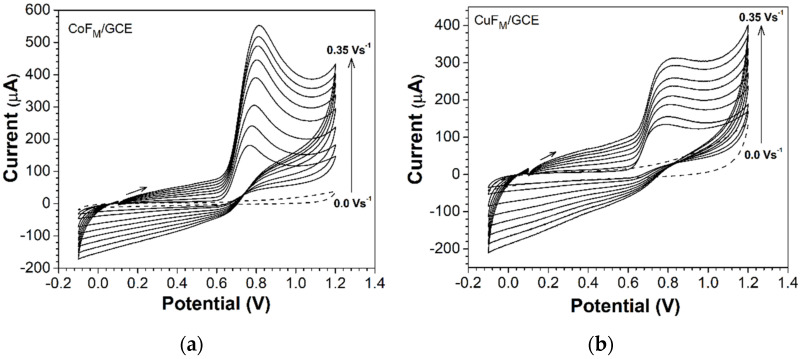
Cyclic voltammograms of 2 × 10^−3^ mol L^−1^ NAP in 0.1 M PBS (pH = 2.5) with (**a**) CoF_M_-modified GCE and (**b**) CuF_M_-modified GCE at different scan rates of from 0.025 at 0.35 Vs^−1^. The dashed line corresponds to the supporting electrolyte at the modified electrode.

The behavior of the anodic peak current vs. the variation of the sweep rate in the range of 25–350 mVs^−1^ is illustrated in Figure 7. A linear trend of the peak current and peak potential according to the scan rate is evident. Furthermore, when the sweep rate was increased, there was an increase in the peak current, and the peak potential shifted toward more positive values, which indicates a diffusion-controlled electrochemical process.

**Figure 8:**
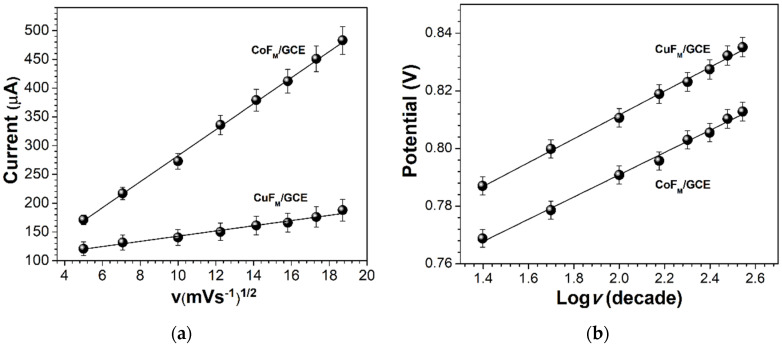
(**a**) Linear plots of peak currents (μA) vs. the square of the scan rate: The CoF_M_ GCE I_pa_ = 22.8642 *v*^1/2^ + 53.58, R^2^ = 0.9983, and the CuF_M_ GCE I_pa_ = 4.6248 *v*^1/2^ + 96.26, R^2^ = 0.9803. (**b**) Linear plots of peak potential vs. the logarithm of the scan rate at the CuF_M_ GCE E_pa_ = 0.04131 Log *v* + 0.7289, R^2^ = 0.9972, and the CoF_M_ GCE E_pa_ = 0.0386 Log *v* + 0.7137, R^2^ = 0.9942 in 0.1-M PBS (pH = 2.5) containing NAP 2 × 10^−3^ mol L^−1^.

The first cycle shows an oxidation peak at 0.786 V for the CuF_M_ GCE and 0.768 V for the CoF_M_ GCE, respectively. Figure 8 contains a linear plot of the peak currents against the square root of the scan rate (v^1/2^) and the potential against the logarithm of the scan rate for both anodic lines (CoF_M_ and CuF_M_ GCEs), thereby confirming a diffusion-controlled process. Figure 8b demonstrates that for the oxidation of NAP using the CoF_M_-modified GCE, lower peak potential values are evident compared with those obtained for the CuF_M_-modified GCE. This could be attributed to the superior catalytic capacity of cobalt over copper in barium hexaferrite.

At each modified electrode the behavior of the anodic peak potential value against the scan rate revealed that the electrochemical process for the anodic oxidation of NAP was irreversible. According to Laviron, peak potential E_p_ for an irreversible electron transfer can be expressed by the following equation:

(1) $$E_{p}{=E}^{0^{\prime }}+\left(\frac{2.30\mathrm{RT}}{\alpha \mathrm{nF}}\right)\log\left(\frac{{\mathrm{RTk}}^{0}}{\alpha \mathrm{nF}}\right)+\left(\frac{2.30\mathrm{RT}}{\alpha \mathrm{nF}}\right)\log\;v,$$

where k^0^ is the standard heterogeneous rate constant of the reaction, E^0′^ is the formal redox potential, α is the charge-transfer coefficient, n is the number of transferred electrons, F is the Faraday constant (96,500 C mol^−1^), and R and T are 8.123 J mol K^−1^ and 298 K, respectively [49]. The value of αn can be calculated easily from the slope of E_p_ vs. the log v plot. The slopes in the given system are for the CuF_M_ GCE (0.0413) and the CoF_M_ GCE (0.0386), respectively. Given that T = 298 K, R = 8.314 J/Kmol, and F = 96,480 C/mol, the value of αn was calculated to be 1.43 (k^0^ = 38.3 s^−1^) and 1.53 (k^0^ = 52.01 s^−1^). In Equation (1), E^0^ was obtained from data provided in an E_p_ vs. v plot at v = 0 (figure not shown); the obtained corresponding E^0^ values for the anodic process observed in Figure 7 were 0.776 V for the CoF_M_ GCE and 0.796 V for the CuF_M_ GCE [49]. The CoF_M_ GCE produced higher αn and k^0^ values, which are associated with the major electroactivity of the CoF_M_ GCE [46,47,48,49]. Then, the αn value was used to calculate n using Equation (2) below:

(2) $$\alpha =\frac{47.7}{E_{p}-\frac{E_{p}}{2}},$$

where E_p/2_ is the half-peak potential, E_p_ is the peak potential, and α is the charge-transfer coefficient [50,51]. The number of exchanged electrons (n) was observed to be ≈ 2, indicating that two electrons were involved in the oxidation of NAP at the CuF_M_ and CoF_M_ GCEs, respectively.

### 2.3. Electroanalysis of Acetaminophen NAP in Acidic Medium

#### 2.3.1. Concentration Analysis of NAP

**Figure 9:**
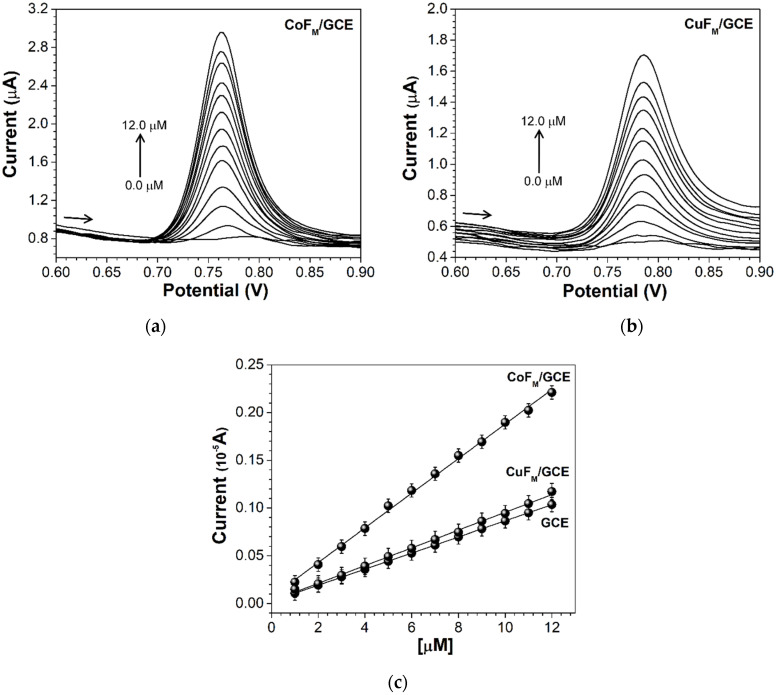
DP voltammograms of acetaminophen at different concentrations (0, 1, 2, 3, 4, 5, 6, 7, 8, 9, 10, 11, and 12 µM, respectively) in 0.1-M PBS (pH = 2.5) solution at the (**a**) CoF_M_ GCE and (**b**) CuF_M_ GCE. (**c**) Linear graph of current peak vs. NAP concentrations. CoF_M_ GCE I_pa_ = 0.181 (NAP) + 0.058, R^2^ = 0.9979, CuF_M_ GCE I_pa_ = 0.0930 (NAP) + 0.0263, R^2^ = 0.9974, and CGE I_pa_ = 0.084 (NAP) + 0.024, R^2^ = 0.9999.

The sensitivity of the method for determining NAP was evaluated using differential pulse voltammetry (DPV). Sharp and well-defined peaks were obtained at lower concentrations of NAP. Typically, the DPV technique is used to quantitatively analyze compounds of pharmaceutical concern, e.g., NAP. DP voltammograms at varying concentrations of NAP are presented in Figure 9a,b. The anodic peak current is seen to increase linearly with concentration in the range of 1.0 × 10^−6^ to 1.2 × 10^−5^ M; Figure 9c shows a linear calibration plot in which each calibration point is the mean of three measurements.

Equation (3) was used to calculate the detection limit (LoD) and the quantification limit (LoQ). The LoD was (0.255 ± 0.005), (0.577 ± 0.007), and (0.595 ± 0.008) µM, while the LoQ was (0.773 ± 0.005), (1.721 ± 0.006), and (1.782 ± 0.004) µM for the CoF_M_, CuF_M_, and GCEs, respectively:

(3) $$\mathrm{LoD}=\frac{3.3\;\times \;\mathrm{SD}}{\mathrm{Slope}}\;\mathrm{and}\;\mathrm{LoQ}=\frac{10.0\;\times \;\mathrm{SD}}{\mathrm{Slope}},$$

where SD is the standard deviation of the peak currents over the slope of the calibrated plot. The nanomaterials have different detection limits, and the LoD of the cobalt-substituted barium hexaferrite is lower than the copper-substituted barium hexaferrite nanomaterial and the bare electrode. These results indicate the excellent electroactivity and greater active area of the cobalt-substituted barium hexaferrite. Accordingly, these results support the feasibility of using M-type barium ferrites in the design of sensing devices for substances of pharmaceutical interest. In this study, the detection of NAP at low concentrations was facilitated using transition metals, such as cobalt.

The LoD obtained in this study compares favorably with those reported in previously published research (see Table 2).

#### 2.3.2. Reproducibility and Stability in the Analysis of NAP with Modified Electrodes

Reproducibility and stability were evaluated using the modified electrode surfaces of the CoF_M_ and CuF_M_ GCEs. The study used 2 × 10^−3^ mol L^−1^ NAP in 0.1-M PBS at pH = 2.50. After 20 consecutive voltammetric cycles ranging from −0.2 to 1.2 V at 25 mVs−^1^, the maximum current decreased by less than 3.2% and 4.4%, respectively; this result indicates that the method provides good reproducibility for the CoF_M_ and CuF_M_ GCEs, respectively [47]. Additionally, the electrodes were unused for 10 days before being utilized in the NAP analysis. According to the CV and DPV responses of both modified electrodes, there was little change in the peak current, indicating considerable stability of both electrodes [56]. The modified electrode surfaces of the CoF_M_ GCE yielded satisfactory results.

#### 2.3.3. Analysis of Enriched and Real Urine Samples

**Figure 10:**
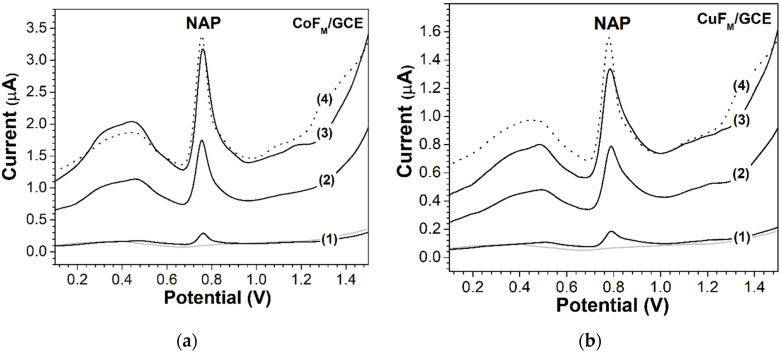
DPVs of acetaminophen NAP prepared in urine media and three samples with additions of (1) 1.0, (2) 5.0, and (3) 10.0 mM. (4) The dotted line indicates 10 µL of a real urine sample 4 h after consumption of SEF. The gray line indicates the absence of NAP. (**a**) CoF_M_/CGE and (**b**) CuF_M_/CGE were modified electrodes respectively.

The modified electrodes were used to quantify NAP concentrations in different pharmaceutical preparations. Additionally, the concentrations of NAP in human urine, which is a more complicated matrix, and commercial acetaminophen tablets were determined. For this investigation, the quantitative determination of NAP in urine samples enriched with commercial medicine containing acetaminophen was studied using DPV. SEVEDOL Extra Strength (SEF), a commercial drug, was selected. This product combines the analgesic properties of acetaminophen with the anti-inflammatory–analgesic activity of ibuprofen; potentiated by the action of caffeine, SEF provides powerful analgesic and anti-inflammatory properties and indicates severe pain, e.g., migraine. The SEF tablets each contained 250, 400, and 65 mg of acetaminophen, ibuprofen, and caffeine. The urine samples were diluted at least 100 times with a PBS buffer (pH = 2.50). The SEF-enriched samples were prepared at the same concentrations that were used to construct the calibration curve (see Figure 10).

It is evident from Figure 10a,b that a wide shoulder occurred at potentials between 0.2 and 0.6 V, which could be associated with the oxidation of ascorbic acid in urine [57]. Additionally, a weak signal around 1.0 V was associated with the oxidation of ibuprofen [58]. Signals corresponding to caffeine were not observed under experimental conditions. According to Figure 10, there is a relative consistency between the behavior of the peak potentials and peak currents with the results obtained in this study up to this point.

The peak potentials in Figure 10a are less positive than the corresponding ones in Figure 10b. The removal of NAP from the capsule supplements before analysis was not necessary.

Voltammetric signals corresponding to the characteristic peak of NAP were observed. The components of the SEF tablet produced no interference in the electrooxidation of NAP. A real human urine sample, which was obtained 4 h after the consumption of SEF, was used to determine the content of excreted NAP; none of the samples were pretreated. The same methodology used for the SEF-enriched samples described above was followed. Figure 10a,b shows the response of the modified electrodes and reveals that both devices responded to the detection of NAP in the actual sample. A calibration chart was used to analyze the enriched NAP in the urine samples; for the CoF_M_ GCE, the recovery ranged from 96.6% to 101.0%, while that of the CuF_M_ GCE was 93.9% to 98.4%. These results demonstrate the outstanding analytical performance of the modified electrode. Table 2 presents the results of the three investigated urine samples; sample 4 (the real urine sample) was analyzed 4 h after the consumption of SEF.

From Table 3, it can be determined that the modified electrodes detected concentrations of SEF in the urine samples according to the content of NAP [59,60,61,62,63]. The NAP concentration obtained in the pharmaceutical formulation agreed with the indicated amount, and the drug content of the tested SEF tablet was within the indicated amount. Therefore, the surface-modified electrodes successfully detected NAP in different samples and matrices.

## 3. Materials and Methods

The chemicals BaCl_2_.2H_2_O, CoCl_2_.2H_2_O, and CuCl_2_.6H_2_O (reagent grade RG) were purchased from Merck, FeCl_3_ was bought from Sigma Aldrich, >98% purity citric acid and C_6_H_8_O_7_ from Merck, RG grade propylene glycol (C_3_H_8_O_2_) and glycerin (C_3_H_8_O_3_) were from Merck. NH_4_OH (RG grade) and acetaminophen (Analytical grade AG) were used as received from Merck. The AG-grade PBS solution (supporting electrolyte) (0.1-M KH_2_PO_4_) was purchased from Merck, and the pH was adjusted using 0.1-M KOH (AG grade, Merck) and H_3_PO_4_ from Sigma Aldrich at a ratio of 1:1. The solutions were prepared using distilled water. The citrate sol gel method was utilized for the synthesis of the transition-metal-substituted barium ferrite nanomaterials, which were characterized using XRD, SEM, Fourier-transform infrared, and Raman analysis techniques [20].

### Electrode Modification and Electrochemical Studies

Substituted M-type barium ferrite nanoparticles (2 mg) were dispersed in 1 mL deionized water before agitation in an ultrasonic bath for 1 h to achieve a well-dispersed suspension. The GCE was polished with 0.05 μm α-Al_2_O_3_, which was rinsed ultrasonically with water and absolute ethanol before sonication in deionized water. The modified electrodes with substituted M-type barium ferrites were prepared by casting 5 μL of the suspension on the surface of the pretreated GCE. Then, the prepared electrodes were maintained at a temperature of 29 °C for 3 h.

The electrochemical measurements were obtained using a computer-controlled AUTOLAB 128N electrochemical analyzer. A double-wall single-compartment cell with a three-electrode configuration was used. The auxiliary and reference electrodes comprised a Pt wire (active surface: d = 0.3 mm, l = 0.7 cm) and Ag/AgCl electrodes, respectively, and all potentials reported in this paper were referred to this reference electrode. The working electrodes comprised a bare GCE with a 3 mm diameter and a modified GCE with substituted M-type barium ferrite nanoparticles (Ba_1.0_Co_1.22_Fe_11.41_O_18.11_ GCE (CoF_M_ GCE) and Ba_1.14_Cu_0.82_Fe_11.65_O_18.02_ GCE (CuF_M_ GCE)). The DPV specifications were 0.1 ≤ E_pa_ ≤ 1.6 V at a step potential of 5 mV, pulse amplitudes of 100 mV, pulse times of 50 ms, and a scan rate of 10 mV s^−1^.

## 4. Conclusions

This study used the authors’ method [20] to prepare substituted barium hexaferrites to modify the surfaces of the electrodes. The use of glycerin produced better than, i.e., propylene glycol and enhanced the formation of hexagonal M-type ferrites. The cobalt- and copper-substituted barium hexaferrite nanomaterials were used to modify the surfaces of GCEs. The electrochemical behavior of the substituted M-type barium ferrite nanoparticles was successfully demonstrated using NAP as a model analyte. NAP oxidation was indicated by the electroactivity of the nano-CoF_M_ GCE and the CuF_M_ GCE. The rate of electron transfer was enhanced by the nanoparticles, and the NAP oxidation current at the modified electrode surfaces was significantly improved. A graph of the peak currents against the square root of the scan rate provided regression values of 0.9983 and 0.9903 for both modifiers, respectively, indicating a diffusion-controlled electrochemical process. However, the calculated detection limits corresponded well with those reported in previous studies. Finally, the CoF_M_ GCE sensor exhibited superior voltammetric behavior than both the CuF_M_ and the unmodified GCE. These findings support the use of the proposed sensor for NAP monitoring in pharmaceutical samples.

## Figures and Tables

**Table 1:** Parameters obtained using CV at the bare and modified electrodes with 2 × 10^−3^ mol L^−1^ NAP in 0.1-M PBS (pH = 2.50).

Surface	Peak Current I_pa_ (µA)	Peak Potential E_pa_ (V)
GCE	96.12 ± 4.73	0.864 ± 0.028
CuF_M_/GCE	131.24 ± 4.51	0.793 ± 0.031
CoF_M_/GCE	201.37 ± 4.22	0.777 ± 0.027

**Table 2:** Comparison of the designed sensor with previously reported sensors for NAP determination.

Modified Electrode	Methods	Linear Range(µM)	Analyte	LoD (µM)	Ref
GCE-arene-ruthenium (II) complex	Amperometry	1.99–31	NAP	0.58	[52]
GCE-PVA-Fe_3_O_4_	CV	0–100	NAP	8	[53]
GCE-hexacyanoferrate(III) intercalated Ni Al LDH	CV	3.0–1500	NAP	0.80	[54]
GCE-MWCNT/TiO_2_	CV	10–120	NAP	11.77	[55]
GCE-Ba_1.0_Co_1.22_Fe_11.41_O_18.11_	DPV	1–12	NAP	0.530	This work
GCE-Ba_1.14_Cu_0.82_Fe_11.65_O_18.02_	DPV	1–12	NAP	0.895	This work

Abbreviations: GCE, glass carbon electrode; NAP, acetaminophen; CV, cyclic voltammetry; DPV, differential pulse voltammetry.

**Table 3:** Determination of NAP in urine samples.

Urine	Added (μM)	^(a)^ Detected (µM)		Recovery (%)		SD ± RSD (%)	
		CoF_M_/GCE	CuF_M_/GCE	CoF_M_/GCE	CuF_M_/GCE	CoF_M_/GCE	CuF_M_/GCE
Sample 1	1	0.98	0.95	98.0	95.0	0.019 ± 1.939	0.032 ± 3.371
Sample 2	5	4.99	4.89	99.8	97.8	0.023 ± 0.460	0.021 ± 0.430
Sample 3	10	10.01	9.84	101.0	98.4	0.021 ± 0.210	0.041 ± 0.417
^(b)^ Sample 4	-	10.56	9.18	-	-	-	-
	10	19.86	18.01	96.6	93.9	0.015 ± 0.076	0.031 ± 0.172

^(a)^ Average of three replicates. ^(b)^ Real urine sample obtained 4 h after the consumption of SEF.

## Data Availability

Not applicable.

## Sample Availability

Samples of the compounds are not available from the authors.
